# Mechanistic study on the reaction of pinB-BMes_2_ with alkynes based on experimental investigation and DFT calculations: gradual change of mechanism depending on the substituent†

**DOI:** 10.1039/d1sc02863d

**Published:** 2021-06-17

**Authors:** Linlin Wu, Chiemi Kojima, Ka-Ho Lee, Shogo Morisako, Zhenyang Lin, Makoto Yamashita

**Affiliations:** Department of Chemistry, The Hong Kong University of Science and Technology Clear Water Bay Kowloon Hong Kong chzlin@ust.hk; Department of Applied Chemistry, Faculty of Science and Engineering, Chuo University 1-13-27 Kasuga, Bunkyo-ku Tokyo 112-8551 Japan; Department of Molecular and Macromolecular Chemistry, Graduate School of Engineering, Nagoya University Furo-cho, Chikusa-ku Nagoya Aichi 464-8603 Japan makoto@oec.chembio.nagoya-u.ac.jp

## Abstract

Transition metal-free direct and base-catalyzed 1,2-diborations of arylacetylenes using pinB-BMes_2_ provided a *syn*/*anti*-isomeric mixture of diborylalkenes. The kinetic analysis showed that the reaction rate and isomer ratio were affected by reaction conditions and substituents on the aryl ring. DFT calculations indicated that direct addition proceeded *via* the interaction of acetylene-π with the BMes_2_ fragment. In contrast, for the base-catalyzed diboration, the previously isolated sp^2^–sp^3^ diborane and borataallene were confirmed as stable intermediates by calculations. The whole reaction pathways can be divided into the Bpin-migration and deprotonation steps, where the borataallene should be considered as a common intermediate. It should be noted that the deprotonation step is reversible and affords the kinetically less favoured isomer under the thermodynamic conditions. As a result, the composition of isomeric products, in the base-catalyzed diboration, is attributed to the small difference of activation barriers between direct and base-catalyzed systems.

## Abstract 2

Combination of kinetic and DFT studies revealed a subtle balance for substituent effect toward the regioselectivity of the product in metal-free and base-catalyzed diboration of arylacetylenes.

## Graphical abstract

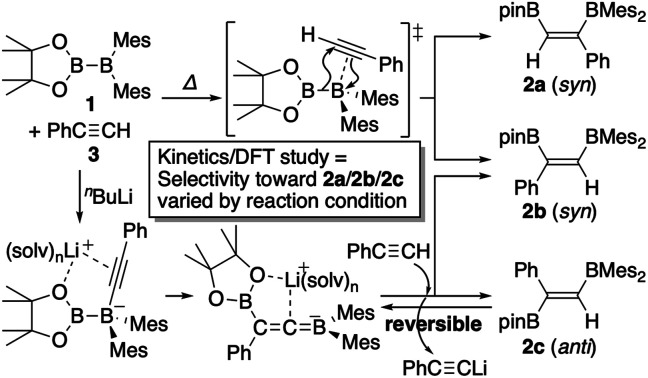

## Introduction

Transition metal-mediated diboration of alkynes to produce diborylalkene is useful to construct tri- and tetra-substituted alkenes in a controlled manner.^1^ In the first report on platinum-mediated diboration of alkynes,^2^ a plausible mechanism was proposed, which consisted of the oxidative addition of the B–B bond of a diborane(4) reagent to Pt, insertion of the alkyne to the Pt–B bond, and B–C bond-forming reductive elimination with experimental observation of a diborylplatinum complex as an intermediate. This observation initiated subsequent mechanistic studies, including isolation of catalytically active intermediates and kinetic analysis.^3^ Thus, the reaction mechanism of transition metal-mediated diboration of alkynes has been well-established.

**Scheme 1:**
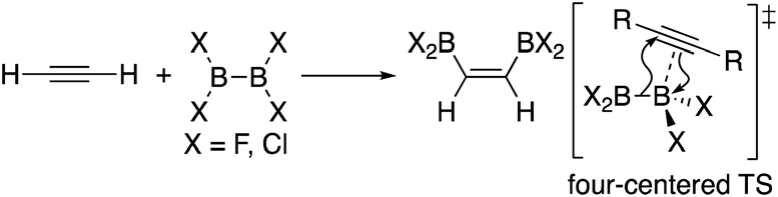
Electrophilic diboration of alkynes by using B_2_X_4_.

**Scheme 2:**
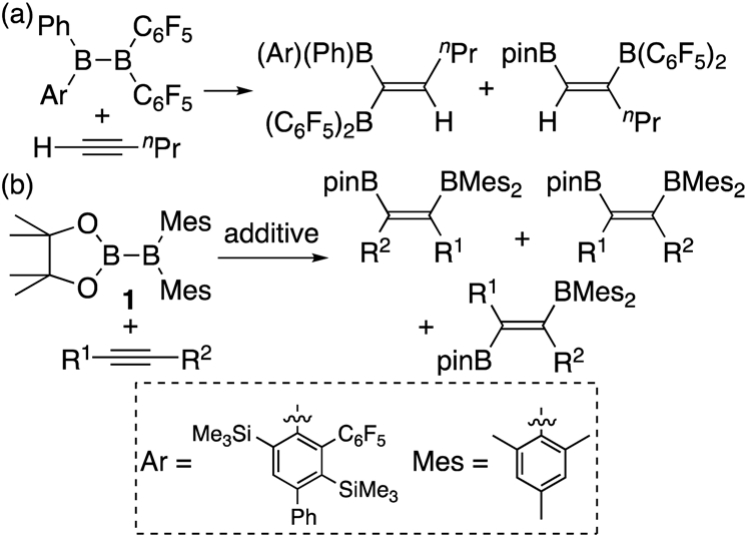
Electrophilic diboration of alkynes by using electron-deficient aryl-substituted diborane(4)s.

In addition to metal-mediated reactions, transition metal-free diborations of alkynes have been reported. The first example was reported for the reaction of acetylene with B_2_F_4_ or B_2_Cl_4_ to produce bis(dihaloboryl)ethylene in 1959 (Scheme 1),^4^ whose four-centered transition state was recently proposed by DFT calculations.^5^ Two seminal reactions, alkoxide-catalyzed diboration of alkenes^6^ and phosphine-catalyzed β-boration of electron-deficient alkenes,^7^ induced the next evolution of the diboration of alkynes using a Lewis base as a catalyst.^8,9^ These reactions involve the formation of sp^2^–sp^3^ intermediates *via* the coordination of the Lewis base to the diborane(4) reagent.^10^ Apart from the development of the base-catalyzed diboration of alkynes, two electrophilic diboration reactions of alkynes were recently reported (Scheme 2). The reaction of tetraaryldiborane(4) with 1-pentyne furnished diborated alkenes as an isomeric mixture (Scheme 2a).^11^ The computational study proposed η^1^-coordinated alkyne complexes as transition states for 1,1- and 1,2-diboration reactions.^12^ We reported the reaction of pinB-BMes_2_ (**1**)^13^ with terminal and internal alkynes resulting in the formation of diborylalkenes (Scheme 2b).^14^ It should be noted that the latter reaction with terminal alkynes could also be catalyzed by using ^*n*^BuLi as an external base, affording different product distributions depending on the reaction conditions.

**Scheme 3:**
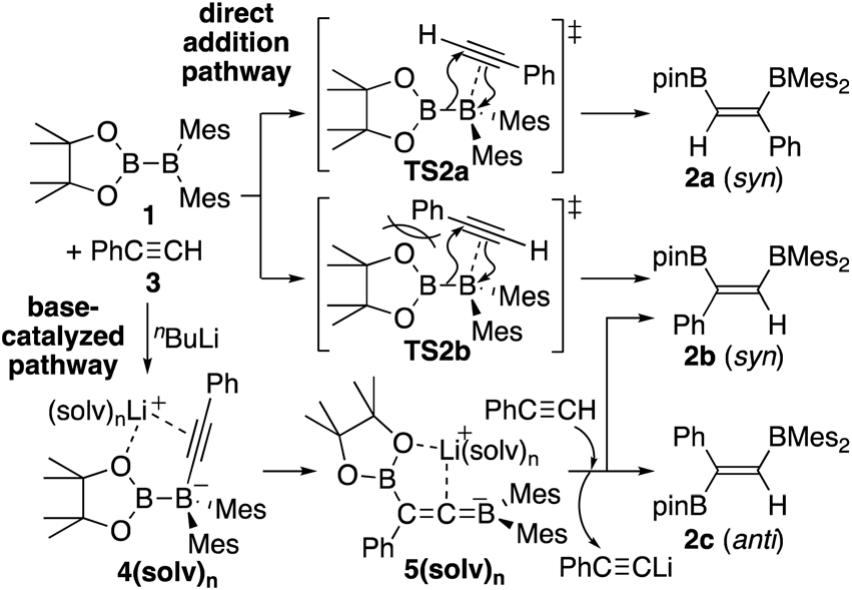
Proposed mechanism for direct and base-catalyzed diboration of phenylacetylene by using pinB-BMes_2_.

In our recent contribution towards the diboration of phenylacetylene derivatives (Scheme 2b) by using pinB-BMes_2_,^14^ we used three different conditions; **A**: simple mixing of the substrate and reagent without any additive, **B**: ^*n*^BuLi-catalyzed diboration, and **C**: ^*n*^BuLi-catalyzed diboration in the presence of a catalytic amount of DME. Considering the formation of three isomers, we tentatively propose a mechanism (Scheme 3) consisting of a concerted pathway through alkyne-coordinating transition states **TS2a** and **TS2b** (direct addition pathway) and a stepwise pathway through two intermediates **4(solv)n** and **5(solv)n** (base-catalyzed pathway), which were independently isolated and structurally characterized.

Although the *anti*-selectivity of the reaction in Scheme 2(b) is remarkable, it was very difficult to understand the substituent effect toward the selectivity for each isomer and reaction rate. Some of the representative results with three phenylacetylene derivatives **3**, **3′**, and **3′′** in the previous report are summarized in Table 1. These results provide the following eight trends: (i) an electron-poor **R** substituent (run 2 *vs.* run 1) lengthened the reaction time and gave a similar selectivity to that in run 1, (ii) an electron-rich **R** substituent (run 3 *vs.* run 1) accelerated the reaction and changed the selectivity toward isomer **b**; (iii) the ^*n*^BuLi-catalyzed reaction (run 4 *vs.* run 1) was accelerated with selectivity toward isomer **b**; (iv) lower temperature for the ^*n*^BuLi-catalyzed reaction (run 5 *vs.* run 4) changed the selectivity toward isomer **c**; (v) reaction in THF solvent can accelerate reaction even under lower temperature (run 6 *vs.* run 4) with selectivity toward isomer **c**; (vi) ^*n*^BuLi/DME-catalyzed reaction (run 7 *vs.* run 4) was further accelerated with a change in the selectivity toward isomer **c**; (vii) an electron-poor **R** substituent under ^*n*^BuLi/DME-catalyzed conditions (run 8 *vs.* run 7) decelerated the reaction but the selectivity was retained; (viii) an electron-rich **R** substituent under ^*n*^BuLi/DME-catalyzed conditions (run 9 *vs.* run 7) accelerated the reaction with lower selectivity. One can expect that the base-catalyzed reactions under condition **B** or **C** (runs 4–9 in Table 1) involve the direct addition pathway, in which an electron-rich substituent would increase the reaction rate, as a background reaction, in addition to the base-catalyzed pathway. In this paper, we report a detailed experimental study with kinetic analysis and theoretical study with DFT calculations to understand the mechanism of the diboration reaction using pinB-BMes_2_ (**1**).

**Table 1:** Direct and base-catalyzed diboration of alkynes with **1**, extracted from the previous work^14^

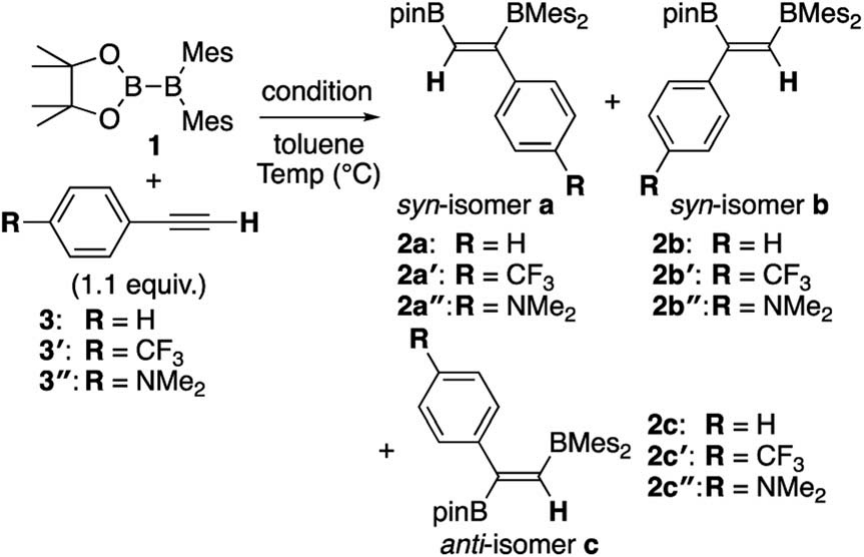							
Run	Condition^a^/solvent	Alkyne	Temp. (°C)	Time (h)	Yield^b^ (%)		
					**a**	**b**	**c**
1	**A**/toluene^c^	**3**	100	37	69	30	
2^c^	**A**/toluene	**3′**	100	59	69	16	
3	**A**/toluene	**3′′**	100	6	24	76	
4	**B**/toluene	**3**	100	17	10	61	8
5	**B**/toluene	**3**	40	42	14	9	58
6	**B**/THF	**3**	70	19	21	7	56
7	**C**/toluene^c^	**3**	100	3	16	9	67
8	**C**/toluene	**3′**	100	8	18	6	59
9	**C**/toluene	**3′′**	100	1.5	13	41	27^d^

a Condition **A**: 1.0 equiv. of **1** (0.4 M), 1.1 equiv. of alkyne. Condition **B**: 1.0 equiv. of **1** (0.2 M), 1.1 equiv. of alkyne, 15 mol% of ^*n*^BuLi. Condition **C**: 1.0 equiv. of **1** (0.4 M), 1.1 equiv. of alkyne, 3 mol% of ^*n*^BuLi and DME.

b NMR yield.

c 0.2 M toluene solution of **1**.

d Not isolated, but tentatively characterized by NMR spectral analysis.

## Results and discussion

### Kinetic study with deuterium labeling

To confirm the relationship among the substituent, the reaction order of **1**, reaction rate, and change of selectivity toward the three isomers **a**, **b**, and **c**, we performed the kinetic analysis of the diboration of terminal alkynes (**3**, **3-d1**, **3′′**, and **3′′-d1**) with **1** under the direct (**A**) and base/DME-catalyzed (**C**) conditions (Table 2). The concentration of **1** was monitored by ^1^H NMR spectroscopy in the presence of excess amount of alkyne to establish a pseudo-first-order reaction. After the reaction was complete, the decay of **1** under each condition was fitted to confirm the first order reaction and to evaluate the observed rate constant, *k*_obs_. The yields of the resulting isomers **a–c** were determined from the integral ratio between the products and internal standard. In run 1 of Table 2, the rate constant for the reaction of **1** with **3** at 100 °C in toluene (same condition as run 1 of Table 1) was estimated as 7.64 ± 0.14 × 10^−4^ (s^−1^) with reproducibility of the isomeric ratio (**a** : **b** = 64% : 32%), which is similar to that in run 1 of Table 1. Changing the solvent to C_6_D_6_ and a lower reaction temperature due to its lower boiling point (run 2) decreased the reaction rate but the isomeric ratio was comparable to that in run 1. Heating **1** with **3-d1** (run 3) gave a slightly larger rate constant than that in run 2, leading to an inverse kinetic isotope effect of 0.93. This result indicates that the reaction involves a concerted pathway with change of hybridization at the acetylenic C–H in the alkyne substrate.^15^ Reaction of **1** with **3′′** (run 4) afforded a larger rate constant than those in runs 2 and 3, indicating the acceleration of the reaction by the introduction of the electron-donating substituent. Simultaneously, the selectivity toward isomer **b** was observed. The reaction with **3′′-d1** (run 5) afforded a slightly smaller rate constant and similar isomeric ratio in comparison with those in run 4, providing a positive KIE of 1.16. Under the base-catalyzed condition **C**, the reaction of **3** (run 6) gave a comparable rate constant and isomeric ratio to those obtained in run 2. The reaction of **3-d1** (run 7) exhibited a smaller rate constant and different selectivity toward the isomer **a** in comparison to those in run 5, leading to a positive KIE of 1.32. In the reaction of **3′′** (run 8), the rate constant is larger than that in run 2, but smaller than that in run 4, with a moderate selectivity toward isomer **b**. The reaction of **3′′-d1** gave a similar rate constant with isomer **b** as the major product (run 9), offering a KIE of 0.99.

**Table 2:** Kinetic analysis of direct or base-catalyzed diboration of alkynes by using **1**

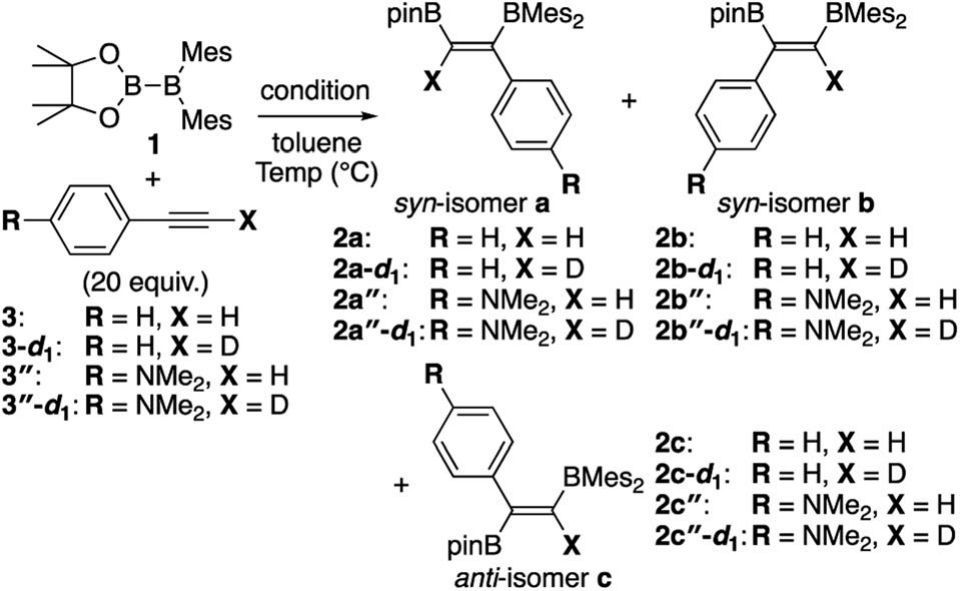								
Run	Condition^a^/solvent	Alkyne	Temp. (°C)	*k*_obs_ b [×10^−4^ s^−1^]	KIE^c^	Yield^d^ (%)		
						**a**	**b**	**c**
1	**A**/tol-*d*_8_	**3**	100	7.64 ± 0.14	—	64	32	—
2	**A**/C_6_D_6_	**3**	70	1.474 ± 0.002	—	65	28	—
3	**A**/C_6_D_6_	**3-d1**	70	1.591 ± 0.003	0.93	70	28	—
4	**A**/C_6_D_6_	**3′′**	70	8.81 ± 0.07	—	19	74	2
5	**A**/C_6_D_6_	**3′′-d1**	70	7.62 ± 0.06	1.16	21	76	3
6	**C**/C_6_D_6_	**3**	70	1.48 ± 0.06	—	36	14	40
7	**C**/C_6_D_6_	**3-d1**	70	1.120 ± 0.003	1.32	48	16	29
8	**C**/C_6_D_6_	**3′′**	70	7.86 ± 0.04	—	18	69	5
9	**C**/C_6_D_6_	**3′′-d1**	70	7.95 ± 0.06	0.99	19	73	5

a Condition **A**: 50 μmol of **1**, 500 μL solvent, 50 μmol of phenanthrene (internal standard); condition **C**: 50 μmol of **1**, 10 mol% of ^*n*^BuLi and DME, 550 μL solvent, 65 μmol of phenanthrene (internal standard).

b Determined by monitoring the decay of **1** with 1st order kinetic analysis.

c KIE: kinetic isotope effect = *k*_H_/*k*_D_.

d ^1^H NMR yield with 1,3,5-trimethoxybenzene as an internal standard (see details in the ESI).

### Elucidation of the reaction mechanism by DFT calculations

In order to elucidate the reaction mechanism, DFT calculations were performed. We first compared the experimentally observed structures with the DFT-calculated structures to see the accuracy of the calculation method. We then studied the direct diboration reaction. Finally, the results of our DFT calculations for the reaction mechanisms of the ^*n*^BuLi-initiated stoichiometric reaction and catalytic diboration are presented.

### Suitability of the calculation method

**Fig. 1:**
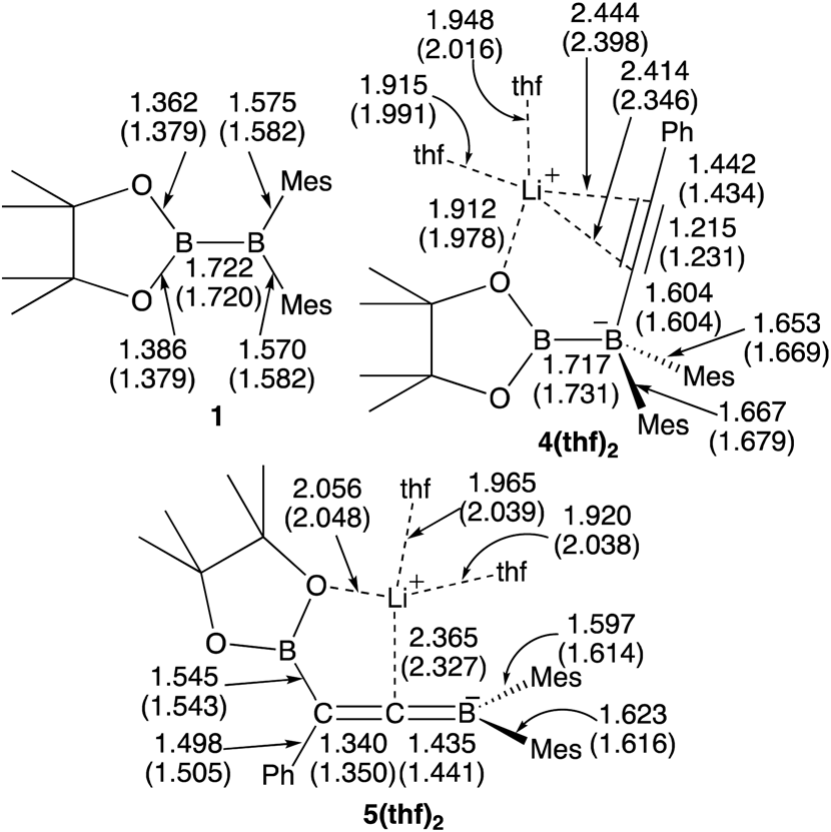
Comparison between the experimental and calculated (in parentheses) structures of **1**, **4(thf)2**, and **5(thf)2**. All the bond distances are given in Å.

We performed calculations with full geometry optimization at the B3LYP-D3/6-31G(d,p) level of theory (see the ESI† for the computational details). All the structural parameters of the starting diborane(4) **1**, the two previously isolated intermediates **4(thf)2** and **5(thf)2**, which were generated by the reaction of **1** with lithium phenylacetylide and subsequent heating,^14^ were well reproduced by DFT calculations (Fig. 1), suggesting that the computational method should give reasonable results.

### Reaction mechanism for the diboration of alkynes in the absence of a base in comparison with the experimental kinetic study

**Fig. 2:**
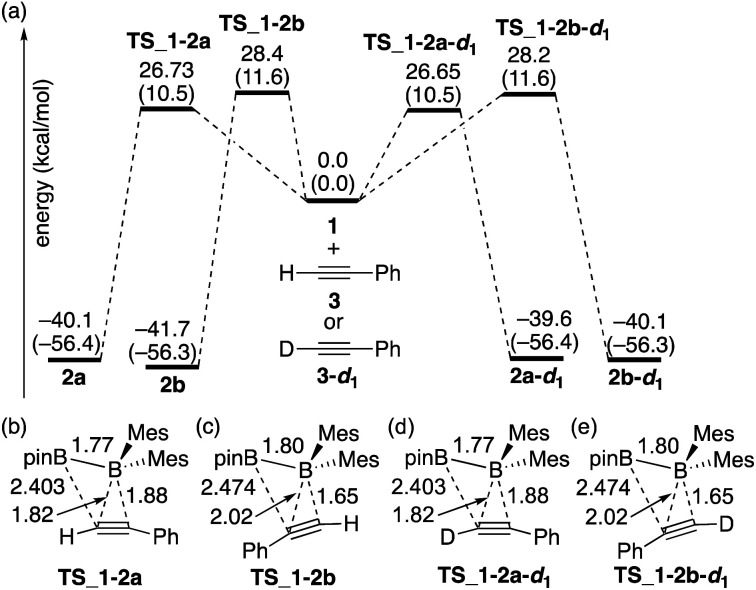
(a) Energy profiles calculated for the direct diboration reactions of **3** and **3-d1** with **1**. The relative free energies and electronic energies (in parentheses) are given in kcal mol^−1^. (b)–(e) Structures of the calculated transition states. Selected bond distances are shown in Å.

The calculated energy profiles for the direct diboration reactions of phenylacetylene (**3**) and phenylacetylene-*d*_1_ (**3-d1**) with diborane(4) **1** under the condition **A** are illustrated in Fig. 2(a). All the calculated transition states show a simple coordination of the alkyne π-bond to the electron-deficient boron center of the BMes_2_ group [Fig. 2(b)–(e)]. The direct diboration of phenylacetylene kinetically favors the transition state **TS_1-2a** over **TS_1-2b** (with the reaction barrier of 26.73 kcal mol^−1^*versus* 28.4 kcal mol^−1^), and that of phenylacetylene-*d*_1_ favors **TS_1-2a-d1** over **TS_1-2b-d1** (with the reaction barrier of 26.65 kcal mol^−1^*versus* 28.2 kcal mol^−1^), consistent with the experimental findings regarding the major and minor products in runs 2 and 3 of Table 2. A slightly lower **TS_1-2b-d1** (26.65 kcal mol^−1^) than **TS_1-2b** (26.73 kcal mol^−1^) would contribute a small inverse KIE, which was observed in the experiment (runs 2 and 3 in Table 2). These results seem consistent with the common notion that an inverse KIE is normally observed for a process involving a change from C(sp) to C(sp^2^).^15^ The calculated energy barriers are over 26.0 kcal mol^−1^ and the calculated reaction energies gained are more than 39.0 kcal mol^−1^, showing that all the reactions are irreversible and the regioselectivity of the reaction is kinetically controlled. It should be noted that the four-centered structures of the transition states in Fig. 2 are similar to those calculated for direct (non-catalyzed) haloboration^16^ and diboration^5,12^ of alkenes and alkynes. Confirmed by intrinsic reaction coordinate (IRC) calculations, the migration of the pinB group to an alkyne carbon proceeds in a concerted fashion, which is consistent with the first order kinetics as was observed in the experiments. Apparently, the orientation of alkyne coordination determines the regioselectivity of the reactions, with the phenyl substituent pointing towards the pinB moiety in diborane being less favored in both cases, indicating that the steric repulsion between pinB and phenyl substituents controlled the selectivity.

**Fig. 3:**
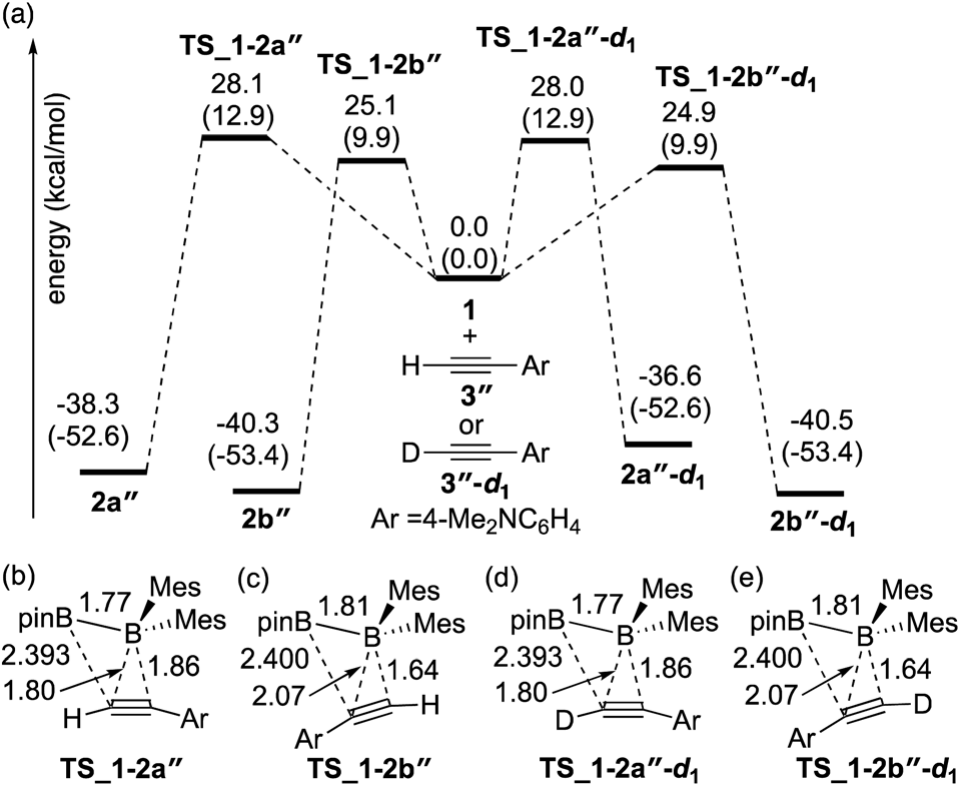
(a) Energy profiles calculated for the direct diboration reactions of **3′′** and **3′′-d1** with **1**. The relative free energies and electronic energies (in parentheses) are given in kcal mol^−1^. (b)–(e) Structures of the calculated transition states. Selected bond distances are shown in Å.

The direct diboration of 4-dimethylaminophenylacetylene **3′′** and its deuterated derivative **3′′-d1** with **1** was also examined as illustrated in Fig. 3(a). Similar to Fig. 2, all the calculated transition states have a four-centered structure [Fig. 3(b)–(e)]. The experimentally observed opposite selectivity (run 4 *vs.* run 2, Table 2) for the formation of isomer **2b′′** could be reproduced by DFT calculations with a lower-energy transition state of **TS_1-2b′′***versus***TS_1-2a′′** (with the reaction barrier of 25.1 kcal mol^−1^*versus* 28.1 kcal mol^−1^). It should be noted that **TS_1-2b′′** is lower in energy than **TS_1-2a**, which is also consistent with the faster reaction rate with **3′′** than with **3**. Transition states **TS_1-2a′′-d1** and **TS_1-2b′′-d1** for the deuterium-labeled substrates were calculated to be almost the same as **TS_1-2a′′** and **TS_1-2b′′** respectively, offering a small KIE value (runs 4 and 5).

### Reaction mechanism for the diboration of phenylacetylene in the presence of a base

#### Evaluation of coordinating solvent

**Scheme 4:**
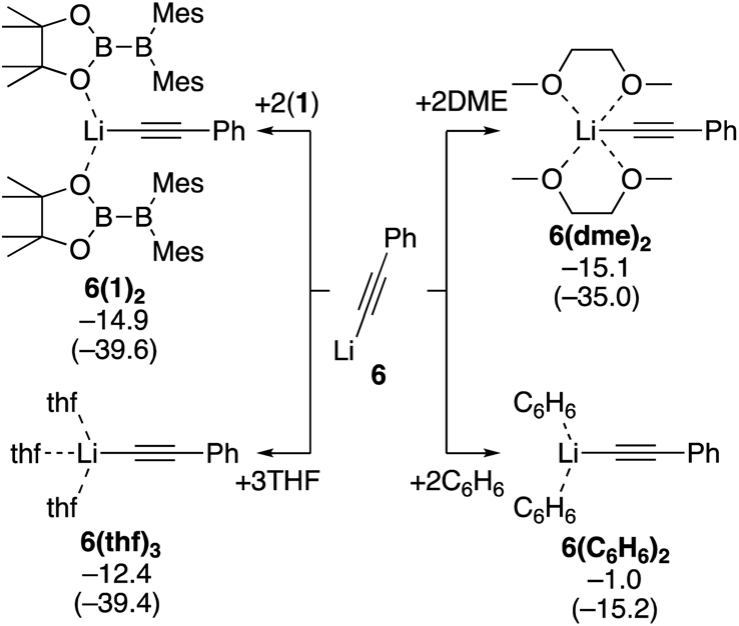
Binding energies of various ligands to PhC≡CLi (**6**). The relative free energies and electronic energies (in parentheses) are given in kcal mol^−1^.

Next, we estimated the mechanism of the diboration of phenylacetylene **3** with **1** under the conditions **B** and **C**. We believe PhC≡CLi (**6**), generated from phenylacetylene and ^*n*^BuLi *in situ*, is the active species that initiates the reactions. Since the coordination environment of a lithium cation depends on the reaction conditions, we first considered how **6** is coordinated to various potential ligands such as the solvent THF and toluene, additive DME, and reactant **1**. We calculated the binding energies of **6** with the different potential ligands mentioned above. Binding energies of all the potential ligands are summarized in Scheme 4. The calculation results show that the two diborane(4) **1** molecules in **6(1)2**, three THF molecules in **6(thf)3**, and two DME molecules in **6(dme)2** coordinate to the lithium center with similar stabilizing energy. In contrast, benzene (representing the actual solvent toluene) is a very poor ligand for the lithium center in **6(C6H6)2**. On the basis of these calculation results, we will consider (i) the use of the diborane(4) reagent **1** as the ligand for the lithium center in the absence of additives and (ii) the use of the solvent THF or additive DME as the ligand when they were employed.

### Reaction mechanism for the base-catalyzed diboration of phenylacetylene (coordinating ligand: diborane(4) 1 = with no ethereal additive)

#### The first half of the reaction: migration of the Bpin group which is the rate-determining step

**Fig. 4:**
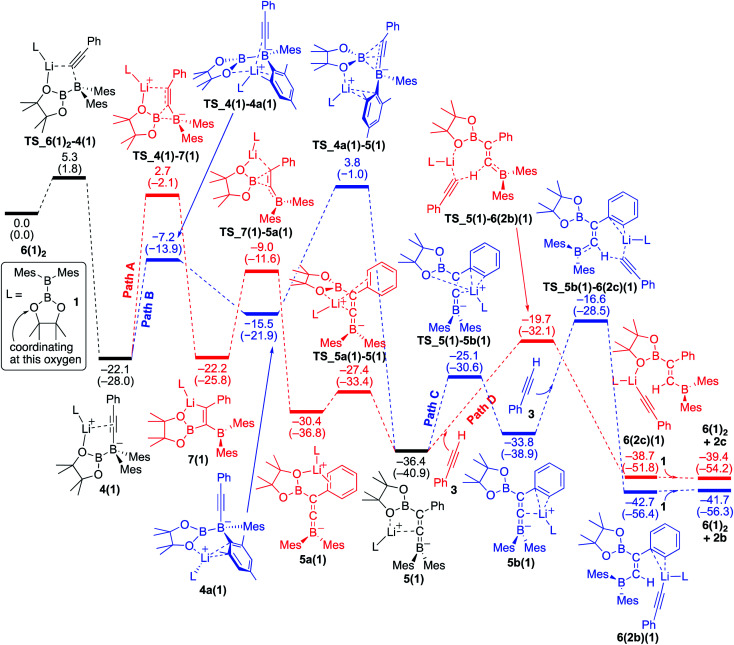
Energy profiles calculated for the base-catalyzed diboration of phenylacetylene with **1** initiated from **6(1)2** in the presence of **1** as the coordinating molecule to the Li^+^ cation. The relative free energies and electronic energies (in parentheses) are given in kcal mol^−1^. Thermodynamically favorable pathways before and after the common intermediate **5(1)** are colored in red.

We conducted DFT calculations where the coordinating ligand to Li^+^ is diborane(4) **1**. As shown in the energy profile (Fig. 4), **6(1)2** undergoes an exothermic PhC≡C^−^ migration from the lithium center to the BMes_2_ boron center with a small barrier of 5.3 kcal mol^−1^, giving **4(1)**. From **4(1)**, there are two pathways [paths A (red) and B (blue)] involving a pinB migration leading to the formation of a common intermediate **5(1)**. Path A consists of the following three steps: (a) a B–B bond cleavage to furnish 1,1-diborylalkeneyllithium **7(1)***via* a ‘1,2-metalate shift’ of alkynylborate,^17^ (b) migration of the pinB group as an intramolecular nucleophilic attack of alkenyllithium to give borataallene **5a(1)**, in which the Li^+^ is coordinated to an oxygen atom from the Bpin moiety and a phenyl ring, and (c) migration of the Li^+^ cation from the phenyl ring to the allenic carbon atom to form the common intermediate **5(1)**,^18^ which is a species similar to the experimentally isolated **5(thf)2** reported in the previous work.^14^ Thus, path A involves two-step migrations of the Bpin substituent. On the other hand, path B consists of two steps: (a) exchange of a ligand coordinating to the Li^+^ from the alkyne to mesityl ring resulting in the formation of **4a(1)**, and (b) a nucleophilic 1,3-migration of the pinB group to a benzylic carbon atom to afford the common intermediate **5(1)**. Our calculations show that these two pathways have comparable reaction barriers, with path A [24.8 kcal mol^−1^*via***TS_4(1)–7(1)**] being slightly more favorable than path B [25.9 kcal mol^−1^*via***TS_4a(1)–5(1)**]. It should be noted that paths A and B here are slightly faster than the direct addition pathway (26.73 kcal mol^−1^) shown in Fig. 2.

#### The second half of the reaction: deprotonation of the acetylene substrate

From the common intermediate **5(1)**, two independent pathways [paths C (blue) and D(red)], which respectively give *syn*- and *anti*-isomers **2b** and **2c** as the products, were found with activation energies of 19.8 and 16.7 kcal mol^−1^. Path C contains the following two steps: (a) migration of the Li^+^ from the oxygen atom of the Bpin group to the phenyl ring to furnish **5b(1)**, and (b) deprotonation of **3**, which is coordinating with lithium, by the allenic carbon of **5b(1)** resulting in the formation of **6(2b)(1)**. The subsequent ligand exchange of **6(2b)(1)** with **1** affords the product **2b** and regenerates **6(1)2** to realize the catalytic cycle. Path D also includes a similar deprotonation of Li^+^-coordinating **3** by the allenic carbon atom of **5(1)** to give **6(2c)(1)**. The subsequent ligand exchange of **6(2c)(1)** with **1** affords the product **2c** and regenerates **6(1)2**. Considering the energy profiles shown in Fig. 4, we see that the rate-determining step of the reaction corresponds to the pinB migration from **4(1)***via* path A with a barrier of 24.8 kcal mol^−1^ at the transition state **TS_4(1)–7(1)**.

#### Reversibility of the deprotonation step that contributes to the selectivity toward the products *syn*-**2b** and *anti*-**2c**

The selectivity towards the formation of **2b** and **2c** is controlled by the deprotonation steps. Under the reaction conditions, the backward reaction of path D is energetically accessible [19.0 kcal mol^−1^, from **6(2c)(1)** to **5(1)**]. Therefore, in the later stage of the catalytic process where the concentration of **1** decreased, this backward reaction and subsequent path C would contribute to produce the *syn*-product **2b**. These calculation results regarding the kinetic and thermodynamic preference are consistent with the experimental observations that **2c** is the major product when the reaction is conducted at 40 °C (Table 1, run 5), while **2b** becomes the major product when the reaction temperature is set to 100 °C (Table 1, run 4).

### Reaction mechanism for the base-catalyzed diboration of alkynes (coordinating ligand: THF)

**Fig. 5:**
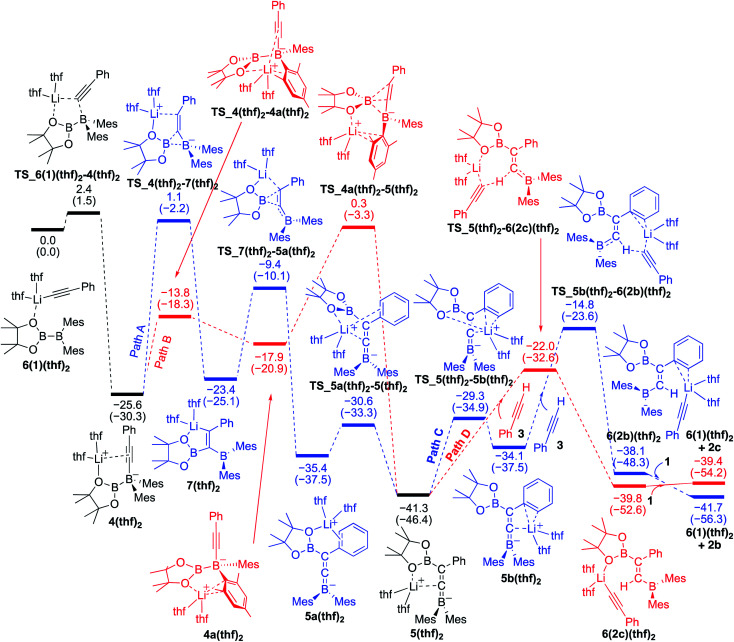
Energy profiles calculated for the base-catalyzed diboration of phenylacetylene with **1** initiated from **6(1)(thf)2** in the presence of THF as the coordinating molecule to the Li^+^ cation. The relative free energies and electronic energies (in parentheses) are given in kcal mol^−1^. Thermodynamically favorable pathways are colored in red.

We also performed calculations for the reaction of diborane **1** in THF solvent. The energy profiles calculated are shown in Fig. 5. The starting compound **6(1)(thf)2** undergoes exothermic alkynyl migration with a small barrier of 2.4 kcal mol^−1^ to give **4(thf)2**, which was directly characterized by the single-crystal X-ray diffraction analysis.^14^ In a similar manner shown in Fig. 4 with **1** as the ligand, two similar pathways were found [paths A (blue) and B (red)]. However, in the case of Fig. 5 with THF as a ligand, the 1,3-migration of pinB *via* path B (25.9 kcal mol^−1^) is kinetically more favorable than the 1,2-migration of pinB *via* path A (26.7 kcal mol^−1^). The results are consistent with the experimental observation that **4(thf)2** was formed at low temperature, and further heating at higher temperature converted **4(thf)2** to **5(thf)2**.^14^ From the common intermediate **5(thf)2**, path D providing the *anti*-product **2c** is kinetically favored by 7.2 kcal mol^−1^ with a smaller activation energy (19.3 kcal mol^−1^) *via***TS_5(thf)2–6(2c)(thf)2** than path C providing the *syn*-product **2b**. The isomerization from **2c** to **2b***via* the common intermediate **5(thf)2** and the transition state **TS_5b(thf)2–6(2b)(thf)2** requires a slightly higher energy of 26.5 kcal mol^−1^ in comparison with that (22.1 kcal mol^−1^) in Fig. 4, suggesting a better product selectivity toward *anti*-isomer **2c** in run 6 (*vs.* runs 4 in Table 1).

### Reaction mechanism for the base-catalyzed diboration of phenylacetylene (coordinating ligand: DME)

**Fig. 6:**
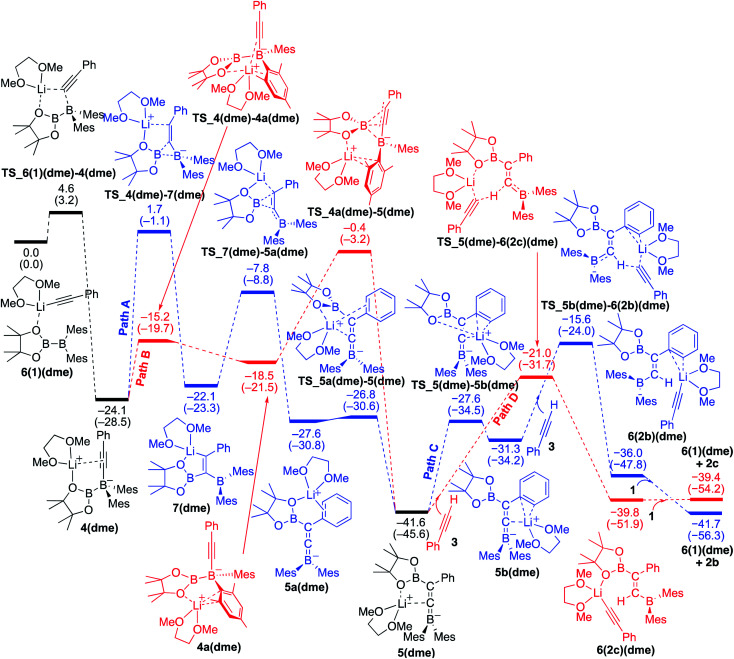
Energy profiles calculated for the base-catalyzed diboration of phenylacetylene with **1** initiated from **6(1)(dme)** in the presence of DME as the coordinating molecule to the Li^+^ cation. The relative free energies and electronic energies (in parentheses) are given in kcal mol^−1^. Thermodynamically favorable pathways are colored in red.

We then considered the situation in which the additive DME acts as a ligand. The corresponding energy profiles calculated on the basis of the mechanisms given are shown in Fig. 6. The property of the obtained energy profile for DME as a ligand in Fig. 6 is almost identical to that of Fig. 5, except that the rate determining barrier [23.7 kcal mol^−1^, which is calculated as the difference between **4(dme)** and **TS_4a(dme)–5(dme)** in path B] is lower than those in Fig. 4 and 5. In other words, DME enhances the reaction rate, consistent with the experimental observation as described in run 7 (*vs.* run 4) of Table 1. The rate enhancement is attributed to the relatively lower isomerization energy from **4(dme)** to **5(dme)** which contributes to the overall energy barrier of path B. The bidentate DME ligands can stabilize the lithium cation to a greater extent than the monodentate ligand **1** and THF, which gives a higher nucleophilicity to the Bpin group and a smaller isomerization energy between intermediates **4a(dme)** and **5(dme)**, and lowers the reaction barrier. Thus, the use of DME as an additive can improve the product selectivity, consistent with the experimental observation.

### Comparison of energy profiles for the base-catalyzed diboration of phenylacetylene, 4-dimethylaminophenylacetylene, and their deuterium-labeled derivatives (coordinating ligand: DME)

**Fig. 7:**
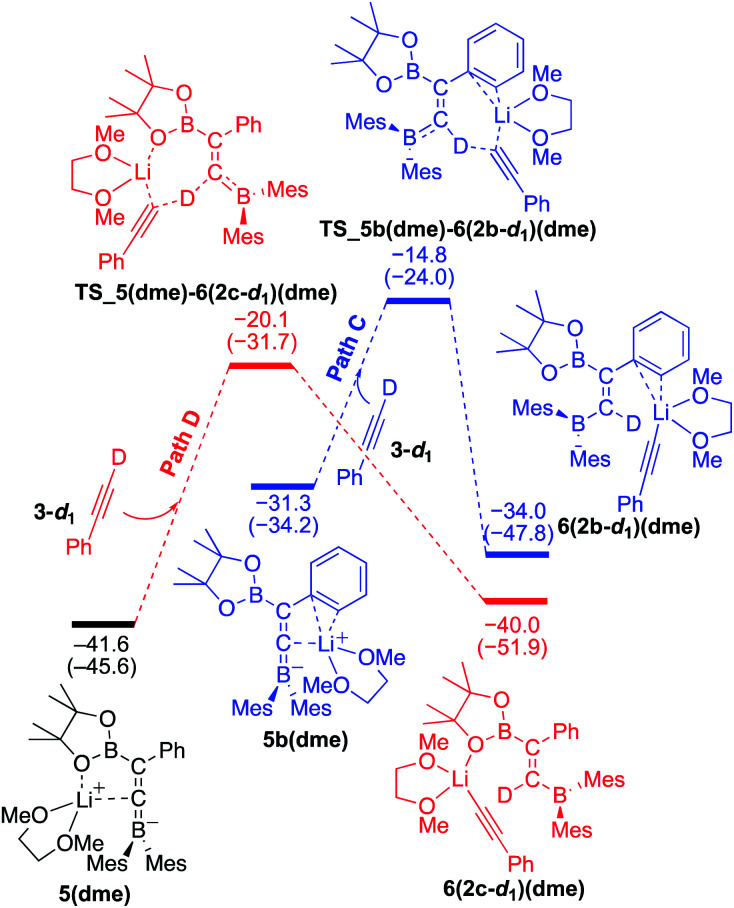
Energy profiles calculated for the paths C and D in the base-catalyzed diboration of phenylacetylene-*d*_1_ with **1** initiated from the common intermediate **5(dme)** (same as Fig. 6) in the presence of DME as the coordinating molecule to the Li^+^ cation. The relative free energies and electronic energies (in parentheses) are given in kcal mol^−1^. Thermodynamically favorable pathways are colored in red.

**Fig. 8:**
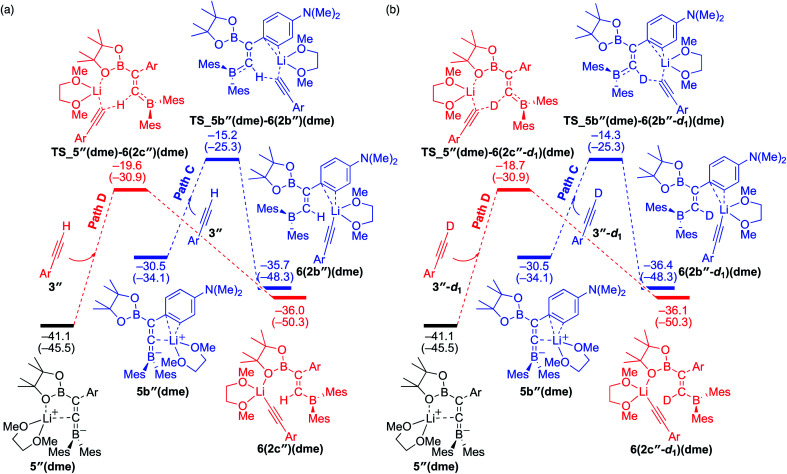
Energy profiles calculated for the paths C and D in the base-catalyzed diboration of **3′′** (a) and **3′′-d1** (b) with **1** initiated from the common intermediate **5′′(dme)** in the presence of DME as the coordinating molecule to the Li^+^ cation. The relative free energies and electronic energies (in parentheses) are given in kcal mol^−1^. Thermodynamically favorable pathways are colored in red.

To understand the results of kinetic experiments in runs 6–9 of Table 2 under base-catalyzed conditions, additional calculations were performed. In runs 6 and 7 of Table 2, deuterium labeling of phenylacetylene led to a slower reaction rate, which was reproduced by DFT calculations for the reaction of the common intermediate **5(dme)** with deuterated phenylacetylene **3-d1** as shown in Fig. 7. The results regarding the kinetic and thermodynamic preference are the same as those in Fig. 6, where path D giving product **2c-d1** is kinetically favored by 5.3 kcal mol^−1^ than path C giving **2b-d1**. Path D in Fig. 7 with **3-d1** requires a slightly higher activation barrier of 21.5 kcal mol^−1^ than that in Fig. 6 with **3** (20.6 kcal mol^−1^), supporting the experimentally obtained positive KIE value. In runs 8 and 9 in Table 2, the reaction rates were almost the same with a KIE value of 0.99 and the selectivities for isomers **a–c** were comparable to each other which were similar to those in runs 4 and 5. The DFT calculations for the reaction between the intermediate **5(dme)** and **3′′-d1** result in the kinetic preference of **2c′′-d1** (path D) to **2b′′-d1** (path C) by 4.4 kcal mol^−1^ (Fig. 8). However, experimentally, no significant KIE was observed. Given the rate-determining activation energy of 23.7 kcal mol^−1^ in Fig. 6 is close to that of **TS_1-2b′′-d1** in Fig. 3 and a lower concentration of catalytically active species for the base-catalyzed condition (10 mol% ^*n*^BuLi and DME were used), the direct diboration of **1** with excess amount of **3′′-d1** is dominant and affords **2b′′-d1** as the major product for runs 8 and 9 in Table 2 even in the presence of catalytic amounts of ^*n*^BuLi and DME.

### Origin of the product distribution in the base-catalyzed diboration of phenylacetylene (Table 1)

**Scheme 5:**
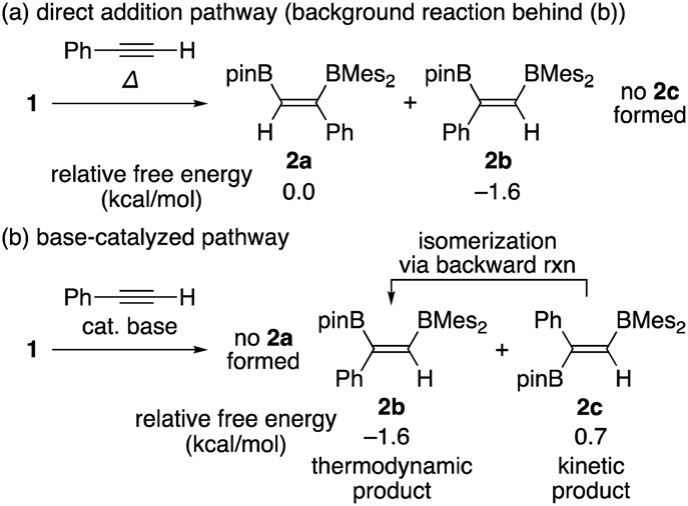
Possible products **2a**, **2b**, and **2c** for the two pathways, relative free energies for each product, and backward isomerization from **2c** to **2b**.

Summarizing the results obtained by DFT calculations, distribution of the isomeric products in the diboration of alkynes by using **1** could be clearly explained by a combination of the direct addition pathway and the base-catalyzed pathway as illustrated in Scheme 5. The DFT calculations indicated that the former reaction affords only **2a** and **2b** [Scheme 5(a)], where the minor product **2b** is thermodynamically more stable, consistent with the irreversible nature of this step. While the base-catalyzed pathway furnished only **2b** and **2c** [Scheme 5(b)], the backward reaction from **2c** to **2b** reflects their relative thermodynamic stability. Therefore, the isomeric mixture of **2a–c** as results of runs 4–9 in Table 1 is expected to originate from both pathways. The composition of the isomeric mixture was also controlled by the difference in the activation barriers for the protonation steps of the borataallene intermediate **5(solv)n** and the relative rate of their backward isomerization as confirmed by our DFT calculations.

## Conclusions

Reaction mechanisms of the transition-metal free direct addition pathway and base-catalyzed pathway in the diborations of arylacetylenes using pinB-BMes_2_ were experimentally and theoretically investigated. For the direct addition pathway, the more Lewis acidic BMes_2_ fragment pulls acetylene to be pyramidalized, followed by migration of the pinB fragment to afford *syn*-adducts **2a** and **2b**. These results indicate that the high Lewis acidity of diborane(4) is the key point for the direct diboration of alkynes. In the presence of ^*n*^BuLi as the base-catalyst, intramolecular 1,3- and/or 1,2-boryl anion migrations take place to afford a common borataallene intermediate, which is converted to *syn*/*anti*-adducts **2b** and **2c**. It should be noted that the kinetically favoured isomer **2c** can be converted to **2b** in the backward reaction. Our experimental and theoretical studies suggest that the reaction barriers of direct addition and base-catalyzed pathways can be controlled by reaction conditions and substituents of arylacetylenes. This knowledge would contribute to further development of selective diboration reactions.

## Data availability

All experimental procedures, analytical data, computational methods, and associated Cartesian coordinates are provided as the ESI.

## Author contributions

LW and CK equally contributed this work. ZL and MY conceived the project. CK and SM conducted all experiments. LW and KHL conducted all DFT calculations. ZL and MY wrote the manuscript. All authors have given approval to the final version of the manuscript.

## Conflicts of interest

There are no conflicts to declare.

## Supplementary Material

SC-012-D1SC02863D-s001

SC-012-D1SC02863D-s002

## References

[cit1] Ishiyama T., Miyaura H. (1999). J. Synth. Org. Chem., Jpn..

[cit2] Ishiyama T., Matsuda N., Miyaura N., Suzuki A. (1993). J. Am. Chem. Soc..

[cit3] Baker R. T., Calabrese J. C., Westcott S. A., Nguyen P., Marder T. B. (1993). J. Am. Chem. Soc..

[cit4] Ceron P., Finch A., Frey J., Kerrigan J., Parsons T., Urry G., Schlesinger H. I. (1959). J. Am. Chem. Soc..

[cit5] Pubill-Ulldemolins C., Fernández E., Bo C., Brown J. M. (2015). Org. Biomol. Chem..

[cit6] Bonet A., Pubill-Ulldemolins C., Bo C., Gulyás H., Fernández E. (2011). Angew. Chem., Int. Ed..

[cit7] Pubill-Ulldemolins C., Bonet A., Gulyas H., Bo C., Fernandez E. (2012). Org. Biomol. Chem..

[cit8] Nagashima Y., Hirano K., Takita R., Uchiyama M. (2014). J. Am. Chem. Soc..

[cit9] Yoshimura A., Takamachi Y., Han L.-B., Ogawa A. (2015). Organosulfide-Catalyzed Diboration of Terminal Alkynes under Light. Chem.–Eur. J..

[cit10] Dewhurst R. D., Neeve E. C., Braunschweig H., Marder T. B. (2015). Chem. Commun..

[cit11] Ge F., Tao X., Daniliuc C. G., Kehr G., Erker G. (2018). Angew. Chem., Int. Ed..

[cit12] Kehr G., Erker G. (2012). Chem. Commun..

[cit13] Asakawa H., Lee K.-H., Lin Z., Yamashita M. (2014). Nat. Commun..

[cit14] Kojima C., Lee K.-H., Lin Z., Yamashita M. (2016). J. Am. Chem. Soc..

[cit15] Anslyn E. V. and Dougherty D. A., Modern Physical Organic Chemistry, University Science Books, 2006

[cit16] Wang C., Uchiyama M. (2012). Eur. J. Org. Chem..

[cit17] Lee M. T., Goodstein M. B., Lalic G. (2019). J. Am. Chem. Soc..

[cit18] Maza R. J., Carbó J. J., Fernández E. (2021). Adv. Synth. Catal..

