# Supplementary material for: Mechanistic study on the reaction of pinB-BMes2 with alkynes based on experimental investigation and DFT calculations: gradual change of mechanism depending on the substituent
Source: Chem Sci. 2021 Jun 17;12(28):9806–15. doi: 10.1039/d1sc02863d (PMC8293998; doi:10.1039/d1sc02863d)
Supplement: SC-012-D1SC02863D-s001 [file SC-012-D1SC02863D-s001.pdf]

Electronic Supplementary Material (ESI) for Chemical Science.
This journal is © The Royal Society of Chemistry 2021

Experimental Section

Synthesis of phenylacetylene-d_1

A Schlenk flask was charged with phenylacetylene (7.00 mL, 63.7 mmol) and hexane (200 mL). The solution was cooled to –78 °C, and then *n*-BuLi (2.6 M, 25.0 mL, 65.0 mmol) was added dropwise to the solution with stirring to induce the immediate formation of white precipitate. After the reaction mixture was slowly warmed to room temperature over 15 h, volatiles were removed from the reaction mixture in vacuo. The precipitate was filtered, washed with hexane, and dried in vacuo to give lithium phenylacetylide as a white powder. The powder was dissolved in Et_2O (50 mL) at –35 °C, then D_2O (15 mL) was added to the reaction mixture. The reaction mixture was extracted with diethyl ether (2×10 mL) and the combined organic phase was evaporated to give a crude product. The crude oil of phenylacetylene-d_1 was distilled by heating at 175 °C under air to give clear oil of phenylacetylene-*d* (3.74 g, 36.2 mmol, 57%), which was characterized by NMR spectroscopy in comparison with those reported in literature.[1]

Synthesis of 4-dimethylaminophenylacetylene-d_1

A Schlenk flask was charged with 4-dimethylaminophenylacetylene (254 mg, 1.75 mmol) and hexane (8 mL). The solution was cooled to –78 °C, and then *n*-BuLi (2.6 M, 750 µL, 2.0 mmol) was added dropwise to the solution with stirring to induce the immediate formation of white precipitate. After the reaction mixture was slowly warmed to room temperature over 14 h, volatiles were removed from the reaction mixture in vacuo. The precipitate was filtered, washed with hexane, and dried in vacuo to give lithium 4-dimethylaminophenylacetylide as a white powder. The powder was dissolved in Et_2O (2 mL) at –35 °C, then D_2O (2 mL) was added the reaction mixture. The reaction mixture was extracted with diethyl ether (2×5 mL) and the combined organic phase was evaporated to give 4-dimethylaminophenylacetylene-d_1 (198 mg, 1.35 mmol, 77%), which was characterized by NMR spectroscopy in comparison with those of protiated compound except alkynyl proton in literature.[2]

Kinetic Study

General remarks

Diborane **1** was used after recrystallization. Phenylacetylene and phenylacetylene-d_1 were used after distillation. 4-Dimethylaminophenylacetylene, 4-dimethylaminophenylacetylene-d_1, and phenanthrene were used after sublimation. All reaction solutions were prepared in an argon-filled glovebox. All reaction rates were estimated by monitoring of decay of **1** in the presence of phenanthrene as an internal standard. A hexane solution of ^{n}BuLi (50.2 µmol) was diluted up to 5.00 mL in a volumetric flask to prepare a 10.0 mM toluene-d_8 or benzene-d_6 solution of ^{n}BuLi. 1,2-dimethoxyethane (DME, 200 µmol) was diluted up to 2.00 mL in a volumetric flask with benzene-d_6 to prepare a 100 mM benzene-d_6 solution of DME.

Condition A (runs 1-5) in Table 2

A 1.00 mL volumetric flask was charged with **1** (37.6 mg, 100 µmol) and phenanthrene (1.0 equiv., 17.9 mg, 100 µmol), followed by diluting up to 1.00 mL with deuterated solvent. An aliquot (500 µL) of the resulting solution was transferred to an NMR tube with J. Young valve. Alkyne (**3**, **3-*d*$_1$**, **3''**, or **3''-*d*$_1$**, 20 equiv., 1.00 mmol) was added to the solution at room temperature. The NMR tube shaken for 1 min and was put into an NMR probe. After the sample was quickly heated to the reaction temperature, locked and shimmed, an integral ratio between **1** and

phenanthrene was monitored by ^{1}H NMR spectroscopy. After the monitoring finished, the ^{1}H NMR yields of the products were estimated by comparison of the integral ratio of the product and internal standard.

Condition B in Table 2

A benzene-d_6 solution of ^{n}BuLi (10.0 mM, 500 µL, 5.00 µmol) was transferred to an NMR tube with J. Young valve, followed by an addition of alkyne (**3**, **3-*d*$_1$**, **3''**, or **3''-*d*$_1$**, 20 equiv., 1.00 mmol). After shaken the NMR tube at room temperature for 3 min, a benzene-d_6 solution of DME (100 mM, 50 µL, 5.00 µmol), **1** (18,8 mg, 50.0 µmol) and phenanthrene (1.3 equiv., 11.7 mg, 65.6 µmol) were added to the reaction mixture at room temperature. The NMR tube shaken for 1 min and was put into an NMR probe. After the sample was quickly heated to the reaction temperature, locked and shimmed, an integral ratio between **1** and phenanthrene was monitored by ^{1}H NMR spectroscopy. After the monitoring finished, the ^{1}H NMR yields of the products were estimated by comparison of the integral ratio of the product and internal standard.

Table S1. Kinetic analysis of direct or base-catalyzed diboration of alkynes with **1**.

	Toluene-d_8 at 100 °C^a	C_6D_6 at 70 °C^a	C_6D_6 at 70 °C, ^{n}BuLi/DME-cat.^{b}
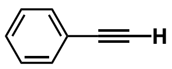	k_{obs} = 7.6±0.1 × 10^{-4}<br>R^2 = 0.9972<br>2981 s, 10 spectra	k_{obs} = 1.474±0.002 × 10^{-4}<br>R^2 = 0.99995<br>14000 s, 29 spectra	k_{obs} = 1.48±0.06 × 10^{-4}<br>R^2 = 0.9980<br>6000 s, 25 spectra
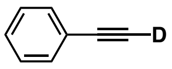	-	k_{obs} = 1.591±0.003 × 10^{-4}<br>R^2 = 0.99992<br>14000 s, 29 spectra	k_{obs} = 1.120±0.003 × 10^{-4}<br>R^2 = 0.9997<br>9750 s, 40 spectra
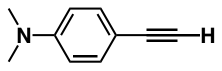	-	k_{obs} = 8.81±0.07 × 10^{-4}<br>R^2 = 0.9993<br>2600 s, 14 spectra	k_{obs} = 7.86±0.04 × 10^{-4}<br>R^2 = 0.9996<br>1950 s, 14 spectra
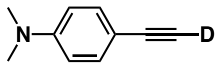	-	k_{obs} = 7.62±0.06 × 10^{-4}<br>R^2 = 0.9994<br>1800 s, 13 spectra	k_{obs} = 7.95±0.06 × 10^{-4}<br>R^2 = 0.9993<br>1950 s, 14 spectra

^{a} 50 µmol of **1**, 20 equiv. of alkyne, 500 µL solvent, 50 µmol of phenanthrene (internal standard); ^{b} 50 µmol of **1**, 20 equiv. of alkyne, 10 mol% of ^{n}BuLi and DME, 550 µL solvent, 65 µmol of phenanthrene (internal standard).

Computational details

The full models for the species involved in the reactions were employed in the calculations. Geometry optimizations were performed without constraints at the Becke 3LYP (B3LYP) level of density functional theory.[3] The 6-31G(d,p) basis set was used to describe the H atom involved in the C–H bond cleavage, 6-31+G(d) basis set was used for Li atom, and the 6-31G(d) basis set was used for all the remaining atoms.[4] Frequency calculations at the same level of theory were performed to verify all the stationary points as minima (zero imaginary frequency) and transition states (one imaginary frequency), and to provide Gibbs free energies. We employed the Gibbs free energies calculated at 298 K for our discussion throughout the paper, considering that entropy cannot be accurately calculated and the contribution of entropy to the free energies will be significantly over-estimated if the actual high temperature is used in the calculation. In the literature, we can find articles employing free energies calculated at 298 K to discuss reactions occurred at other temperatures.[5]

To reduce the overestimation of entropy contribution in the gas-phase results, corrections of −2.6 kcal/mol (or +2.6 kcal/mol) in free energies were made for 2-to-1 (or 1-to-2) transformations.[6] To take dispersion and solvation effects into account, we carried out single-point energy calculations at the B3LYP-D3/6-311+G(d,p) level with the B3LYP optimized structures,[7] employing a continuum medium using UAKS radii on the conductor-like polarizable continuum model (CPCM).[8] Toluene was employed as the solvent in the CPCM calculations according to the reaction conditions. All the calculations were performed with Gaussian 09 (rev. D.01) package.[9] Unless specifically noted, the entropy- and solvation-corrected free energies were used in all of the discussions.

References

1. Weiss, C. J.; Marks, T. J. Organozirconium Complexes as Catalysts for Markovnikov-Selective Intermolecular Hydrothiolation of Terminal Alkynes: Scope and Mechanism. *J. Am. Chem. Soc.* **2010,** *132*, 10533-10546.
2. Yang, J.; Dass, A.; Rawashdeh, A.-M. M.; Sotiriou-Leventis, C.; Panzner, M. J.; Tyson, D. S.; Kinder, J. D.; Leventis, N. Arylethynyl Substituted 9,10-Anthraquinones: Tunable Stokes Shifts by Substitution and Solvent Polarity. *Chem. Mater.* **2004,** *16*, 3457-3468.
3. (a) Lee, C.; Yang, W.; Parr, R. G. Development of the Colle-Salvetti correlation-energy formula into a functional of the electron density. *Phys. Rev. B* **1988,** *37*, 785-789; (b) Becke, A. D. Density-functional exchange-energy approximation with correct asymptotic behavior. *Phys. Rev. A* **1988,** *38*, 3098-3100; (c) Becke, A. D. Density‐functional thermochemistry. III. The role of exact exchange. *J. Chem. Phys.* **1993,** *98*, 5648-5652.
4. Huzinaga, S.; Andzelm, J.; Klobukowski, M.; Radzio-Andzelm, E.; Sakai, Y.; Tatewaki, H., *Gaussian basis sets for molecular calculations*. Elsevier: 1984.
5. (a) Helmstedt, U.; Clot, E. Hydride Ligands Make the Difference: Density Functional Study of the Mechanism of the Murai Reaction Catalyzed by [Ru(H)2(H2)2(PR3)2] (R=cyclohexyl). *Chem. Eur. J.* **2012,** *18*, 11449-11458; (b) Liu, F.; Paton, R. S.; Kim, S.; Liang, Y.; Houk, K. N. Diels–Alder Reactivities of Strained and Unstrained Cycloalkenes with Normal and Inverse-Electron-Demand Dienes: Activation Barriers and Distortion/Interaction Analysis. *J. Am. Chem. Soc.* **2013,** *135*, 15642-15649; (c) Xu, L.; Chung, L. W.; Wu, Y.-D. Mechanism of Ni-NHC Catalyzed Hydrogenolysis of Aryl Ethers: Roles of the Excess Base. *ACS Catalysis* **2016,** *6*, 483-493; (d) Wang, Z.; Zhou, Y.; Lam, W. H.; Lin, Z. DFT Studies of Ru-Catalyzed C–O versus C–H Bond Functionalization of Aryl Ethers with Organoboronates. *Organometallics* **2017,** *36*, 2354-2363.
6. Benson, S. W., *The Foundations of Chemical Kinetics*. Krieger: Malabar, FL, 1982.
7. Grimme, S.; Antony, J.; Ehrlich, S.; Krieg, H. A consistent and accurate ab initio parametrization of density functional dispersion correction (DFT-D) for the 94 elements H-Pu. *J. Chem. Phys.* **2010,** *132*, 154104.
8. (a) Cossi, M.; Barone, V.; Cammi, R.; Tomasi, J. Ab initio study of solvated molecules: a new implementation of the polarizable continuum model. *Chem. Phys. Lett.* **1996,** *255*, 327-335; (b) Cossi, M.; Barone, V.; Mennucci, B.; Tomasi, J. Ab initio study of ionic solutions by a polarizable continuum dielectric model. *Chem. Phys. Lett.* **1998,** *286*, 253-260; (c) Cossi, M.; Barone, V.; Robb, M. A. A direct procedure for the evaluation of solvent effects in MC-SCF calculations. *J. Chem. Phys.* **1999,** *111*, 5295-5302; (d) Cossi, M.; Rega, N.; Scalmani, G.; Barone, V. Energies, structures, and electronic properties of molecules in solution with the C-PCM solvation model. *J. Comput. Chem.* **2003,** *24*, 669-681.
9. Frisch, M. J.; Trucks, G. W.; Schlegel, H. B.; Scuseria, G. E.; Robb, M. A.; Cheeseman, J. R.; Scalmani, G.; Barone, V.; Mennucci, B.; Petersson, G. A.; Nakatsuji, H.; Caricato, M.; Li, X.; Hratchian, H. P.; Izmaylov, A. F.; Bloino, J.; Zheng, G.; Sonnenberg, J. L.; Hada, M.; Ehara, M.; Toyota, K.; Fukuda, R.; Hasegawa, J.; Ishida, M.; Nakajima, T.; Honda, Y.; Kitao, O.; Nakai, H.; Vreven, T.; Montgomery Jr, J. A.; Peralta, J. E.; Ogliaro, F.; Bearpark, M. J.; Heyd, J.; Brothers, E. N.; Kudin, K. N.; Staroverov, V. N.; Kobayashi, R.; Normand, J.; Raghavachari, K.; Rendell, A. P.; Burant, J. C.; Iyengar, S. S.; Tomasi, J.; Cossi, M.; Rega, N.; Millam, N. J.; Klene, M.; Knox, J. E.; Cross, J. B.; Bakken, V.; Adamo, C.; Jaramillo, J.; Gomperts, R.; Stratmann, R. E.; Yazyev, O.; Austin, A. J.; Cammi, R.; Pomelli, C.; Ochterski, J. W.; Martin, R. L.; Morokuma, K.; Zakrzewski, V. G.; Voth, G. A.; Salvador, P.; Dannenberg, J. J.; Dapprich, S.; Daniels, A. D.; Farkas, Ö.; Foresman, J. B.; Ortiz, J. V.; Cioslowski, J.; Fox, D. J., *Gaussian09, Revision D.01*. Gaussian, Inc.: Wallingford, CT, 2009.
